# Genetic profiles of ten *Dirofilaria immitis* isolates susceptible or resistant to macrocyclic lactone heartworm preventives

**DOI:** 10.1186/s13071-017-2428-6

**Published:** 2017-11-09

**Authors:** Catherine Bourguinat, Kathy Keller, Jianguo Xia, Pierre Lepage, Tom L. McTier, Debra J. Woods, Roger K. Prichard

**Affiliations:** 1 Institute of Parasitology, McGill University, 21111 Lakeshore Road, Sainte Anne de Bellevue, QC H9X3V9 Canada; 2 Department of Animal Science, McGill University, Sainte Anne de Bellevue, QC Canada; 3 McGill University and Génome Québec Innovation Centre, Montréal, QC Canada; 4 Zoetis, Veterinary Medicine Research and Development, Kalamazoo, MI USA

**Keywords:** *Dirofilaria immitis*, Heartworm, Macrocyclic lactone resistance, Genetic markers, Predictive model

## Abstract

**Background:**

For dogs and cats, chemoprophylaxis with macrocyclic lactone (ML) preventives for heartworm disease is widely used in the United States and other countries. Since 2005, cases of loss of efficacy (LOE) of heartworm preventives have been reported in the U.S. More recently, ML-resistant *D. immitis* isolates were confirmed. Previous work identified 42 genetic markers that could predict ML response in individual samples. For field surveillance, it would be more appropriate to work on microfilarial pools from individual dogs with a smaller subset of genetic markers.

**Methods:**

MiSeq technology was used to identify allele frequencies with the 42 genetic markers previously reported. Microfilaria from ten well-characterized new isolates called ZoeKY, ZoeMI, ZoeGCFL, ZoeAL, ZoeMP3, ZoeMO, ZoeAMAL, ZoeLA, ZoeJYD-34, and Metairie were extracted from fresh blood from dogs. DNA were extracted and sequenced with MiSeq technology. Allele frequencies were calculated and compared with the previously reported susceptible, LOE, and resistant *D. immitis* populations.

**Results:**

The allele frequencies identified in the current resistant and susceptible isolates were in accordance with the allele frequencies previously reported in related phenotypes. The ZoeMO population, a subset of the ZoeJYD-34 population, showed a genetic profile that was consistent with some reversion towards susceptibility compared with the parental ZoeJYD-34 population. The Random Forest algorithm was used to create a predictive model using different SNPs. The model with a combination of three SNPs (NODE_42411_RC, NODE_21554_RC, and NODE_45689) appears to be suitable for future monitoring.

**Conclusions:**

MiSeq technology provided a suitable methodology to work with the microfilarial samples. The list of SNPs that showed good predictability for ML resistance was narrowed. Additional phenotypically well characterized *D. immitis* isolates are required to finalize the best set of SNPs to be used for large scale ML resistance screening.

**Electronic supplementary material:**

The online version of this article (10.1186/s13071-017-2428-6) contains supplementary material, which is available to authorized users.

## Background

*Dirofilaria immitis* is the causative agent of heartworm disease, which can produce life-threatening morbidity that affects dogs, cats, and wild canids [1–6]. This filarial nematode is distributed in North and South America, Southern Europe, Japan, Australia, India, and China [7, 8]. The macrocyclic lactones (ML) milbemycin oxime, ivermectin, moxidectin, and selamectin are the available prophylactic drugs in the U.S. veterinary marketplace that prevent the establishment of L3–L4 larval *D. immitis* stages in dogs and cats [9]. The first ML loss of efficacy (LOE) report was published in 2005 [10]. Reports of LOE dogs in the United States have persisted over the years. Some of these suspected LOE cases are no doubt due to lack of full compliance with recommended chemoprophylaxis regimens [11]. Nevertheless, recently ML resistance has been confirmed in the U.S. [12, 13]. Because ML resistance has a genetic origin [14], whole genome analysis has been performed on well characterized susceptible *D. immitis* isolates from the U.S., Italy, Gran Canaria, and Grenada and LOE isolates from the U.S. to identify genetic differences that could correlate with evidence of LOE and resistance [13]. One hundred eighty-six single nucleotide polymorphisms (SNP) showed highly significant differences between pools of susceptible and LOE *D. immitis*. Based on these 186 SNPs, Sequenom® SNP frequency analyses were conducted on 663 individual parasites (adult worms and microfilariae) which were phenotypically characterized as susceptible (SUS), confirmed ML treatment survivors/resistant (RES), or suspected resistant/loss of efficacy (LOE) parasites. This approach identified 42 SNPs that appeared to differentiate ML-susceptible from LOE and resistant *D. immitis* isolates [13]. It is highly desirable to reduce the number of marker SNPs using additional ML phenotypically characterized *D. immitis* isolates. Previously, only a small number of confirmed resistant isolates had been genetically characterized. Ultimately, the goal is to build a robust protocol for field application that could be used to monitor for resistant *D. immitis* isolates in the field. Such genetic markers for susceptibility/resistance may also be useful in developing protocols for managing drug resistance in *D. immitis* and for establishing improved scientifically based protocols for registration of new heartworm preventives.

## Methods

### Samples

Ten isolates were provided by Zoetis Animal Health for analysis. The detailed information on the isolates has been published elsewhere [15, 16]. The ML phenotype response was assessed in efficacy studies with a dose of 3 μg/kg of moxidectin (MOX), and the origin of the isolates are presented in Table 1. In total, five isolates were susceptible to MOX while five were resistant to heartworm preventive to varying degrees.

**Table 1:** *Dirofilaria immitis* isolates used for MiSeq sequencing

Isolate	Efficacy percentage reduction^a^ (%)	Phenotype	Origin of the Isolate in USA
ZoeMI	100	Susceptible	MI
ZoeGCFL	100	Susceptible	Fort Myers, FL.
ZoeAL	100	Susceptible	Westover, AL
ZoeKY	100	Susceptible	Salyersville, KY
ZoeMP3	100	Susceptible^b^	TRS Lab, GA
ZoeMO^c^	82	Resistant	Pittsfield, IL/Keytesville, MO/Stanwood, MI
ZoeAMAL	62	Resistant	Westover, AL
ZoeLA	54	Resistant	Ellis, AR/Slaughter, LA
ZoeJYD-34	19	Resistant	Pittsfield, IL/Keytesville, MO/Stanwood, MI
Metairie^d^	N/A	Resistant	Metairie, LA and spent time in MS

^a^Information related to moxidectin phenotypic response is from McTier et al. [15]. The efficacy study was based on 3 μg/kg of oral moxidectin. Efficacy studies described in McTier et al. [15]

^b^Predominantly susceptible.

^c^Relative of ZoeJYD-34

^d^Information on the resistant Metairie isolate is available in Maclean et al. [16]

### Sample manipulation and DNA extraction

Microfilariae (MF) were shipped to McGill University in fresh blood collected in EDTA tubes from untreated dogs infected with each isolate. A filtration procedure [13] was used to extract and clean MF from a 15- to 20-mL blood sample. Polycarbonate membrane filters (3.0 μm; 25 mm; Sterlitech® Corporation, Kent, WA, USA) were used for the filtration. A 1:1 dilution from venous blood with NaHCO_3_ (SIGMA®, Aldrich Co., Oakville, ON, Canada) solution (2 g/L) was made before filtration (5–20 mL per filter). DNA extraction of pooled MF was achieved using QIAamp® DNA Micro kit (Qiagen Inc., Toronto, ON, Canada). DNA concentrations for all samples were evaluated using the Quant-iT™ PicoGreen DNA Assay Kit (Invitrogen®, Life Technologies Inc., Burlington, ON, Canada).

### SNP markers

Based on previous work, 42 SNPs out of the 186 identified from the whole genome [13] were evaluated as they seemed to better differentiate the ML-susceptible phenotype from the LOE and resistant phenotype. The list of SNP positions investigated in the current study is available in Additional file 1 at the end of this article.

### Genetic analysis

Ten DNA pools of MF were sequenced with MiSeq® at Génome Québec Innovation Centre (McGill University). Libraries were prepared following two successive thermocycler steps for tagging with CS1 and CS2 primers [17] and barcoding the fragments. The lists of primers are available in Additional file 2 at the end of this article. Then the fragments were pooled and purified using AMPure XP beads (Beckman Coulter, Inc.) [18], and library quality control was performed. Libraries were then run on a MiSeq® sequencing system using paired end read of 250 base pairs (PE250).

### Data analysis

Reads obtained from MiSeq® were trimmed from the 3’ end to have a Phred score of at least 30. Illumina sequencing adapters were removed from the reads, and all reads were required to have a length of at least 50 bp. Trimming and clipping were performed using Trimmomatic (http://www.usadellab.org/cms/?page=trimmomatic) [19]. The filtered reads were aligned to the nDi.2.2. *D. immitis* genome (http://www.nematodes.org/genomes/dirofilaria_immitis/). Each read set was aligned using BWA (http://bio-bwa.sourceforge.net/) [20], which has a low error rate (<3%), and which a Binary Alignment Map file (.bam) created. Then, all read set BAM files from the same sample were merged into a single global BAM file using Picard (http://broadinstitute.github.io/picard/). BVATool (https://bitbucket.org/mugqic/bvatools/src) was then used to extract from the BAM files the read frequencies at each of the 42 SNPs. The read frequencies were assimilated to the allele frequencies. Allele frequencies from the ten isolates were compared with those of isolates described previously [13].

### Statistical analysis

The identification of the best SNPs to predict ML resistance from these samples was assessed visually by plotting the allele frequencies using GraphPad Prism Software (Version 5). Any allele frequency difference between groups was assessed using Chi-squared tests. The limit of significance was *p* value = 0.05.

### Predictive model

The Random Forest algorithm [21] as implemented in the “Biomarker analysis” module in MetaboAnalyst 3.0 (http://www.metaboanalyst.ca) [22–26] was used to build classification models and to evaluate their performance in predicting ML phenotypes in *D. immitis*. Random Forest is a well-established algorithm based on ensemble learning using a multitude of decision trees. It has been successfully used in building predictive models from SNP data [27, 28]. The tool allowed the identification of different SNP combinations that could best distinguish the two groups. In this case, a score of zero was the optimal value for ML susceptibility prediction; and a score of one was the optimal value for ML resistance prediction. Using a cut-off of 0.5, any sample with a predicted class probability less than 0.5 was considered ML susceptible while any sample with a predicted class probability higher than 0.5 was considered ML resistant. The sensitivity [True Positive/(True Positive + False Negative)] and the specificity [True Negative/(False Positive + True Negative)] of the different models based on different numbers of SNP combinations were obtained using MetaboAnalyst 3.0. To obtain further insight, a heat map was constructed with the percentage of the alternative allele that characterized resistance and the phenotype response from the ML efficacy study of each isolate. The Metairie isolate was not used in the heat map, as the percentage efficacy was not known, although the isolate was classified as resistant. The susceptible samples from a previous study [13] were assumed to give 100% reduction in an efficacy trial, while RES-1 and RES-2, a resistant isolate [13], were known to have 21.6% and 71.3% efficacy, respectively, with ivermectin.

## Results

### Genetic analysis

**Fig. 1:**
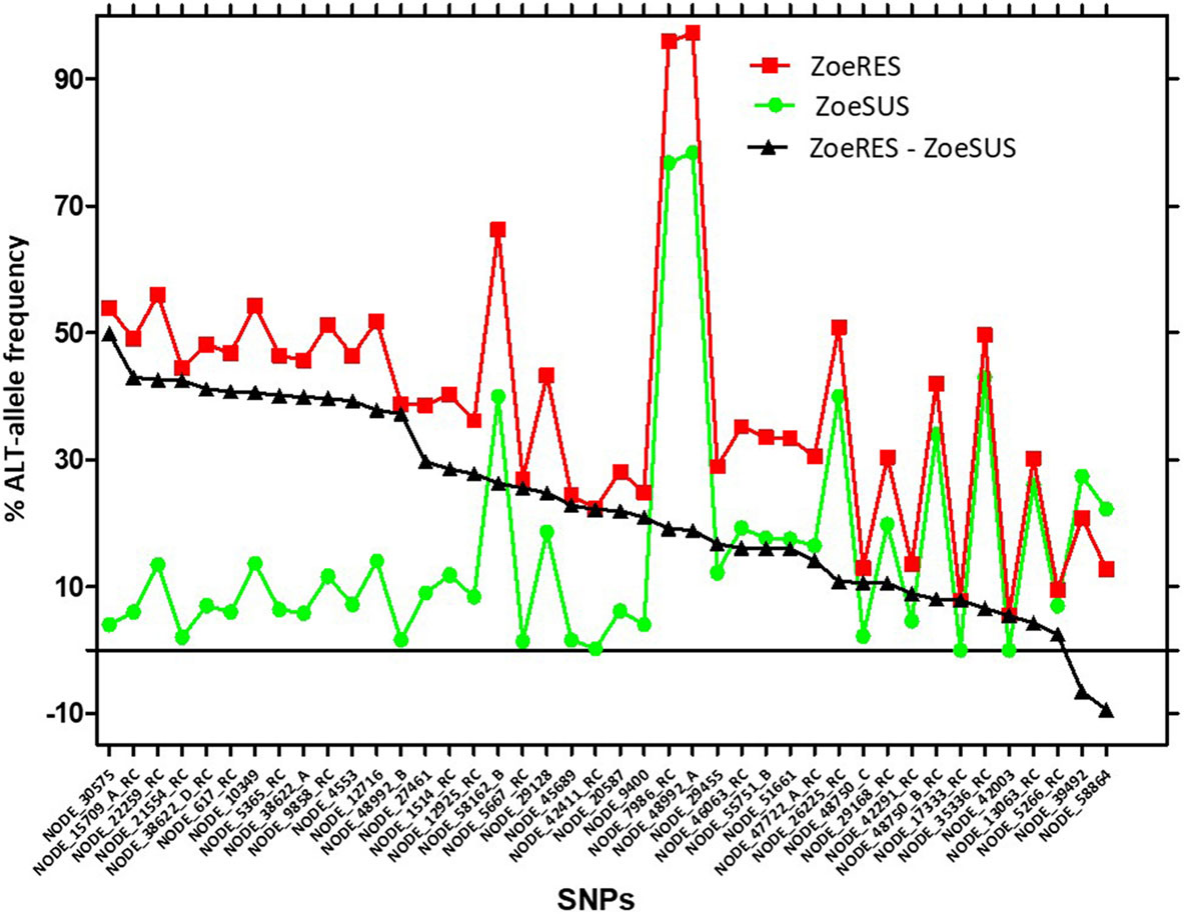
Percentage frequencies of the alternative alleles at 42 previously reported SNP positions in ZoeSUS and ZoeRES isolates. ZoeSUS correspond to ML-susceptible isolates (ZoeKY, ZoeMI, ZoeGCFL, ZoeAL, and ZoeMP3). ZoeRES correspond to MLresistant isolates (ZoeMO, ZoeAMAL, ZoeLA, ZoeJYD-34, and Metairie). ZoeRES – ZoeSUS is the difference in the percentage alternative allele frequencies between ZoeRES and ZoeSUS. RC stands for reverse complement

**Fig. 2:**
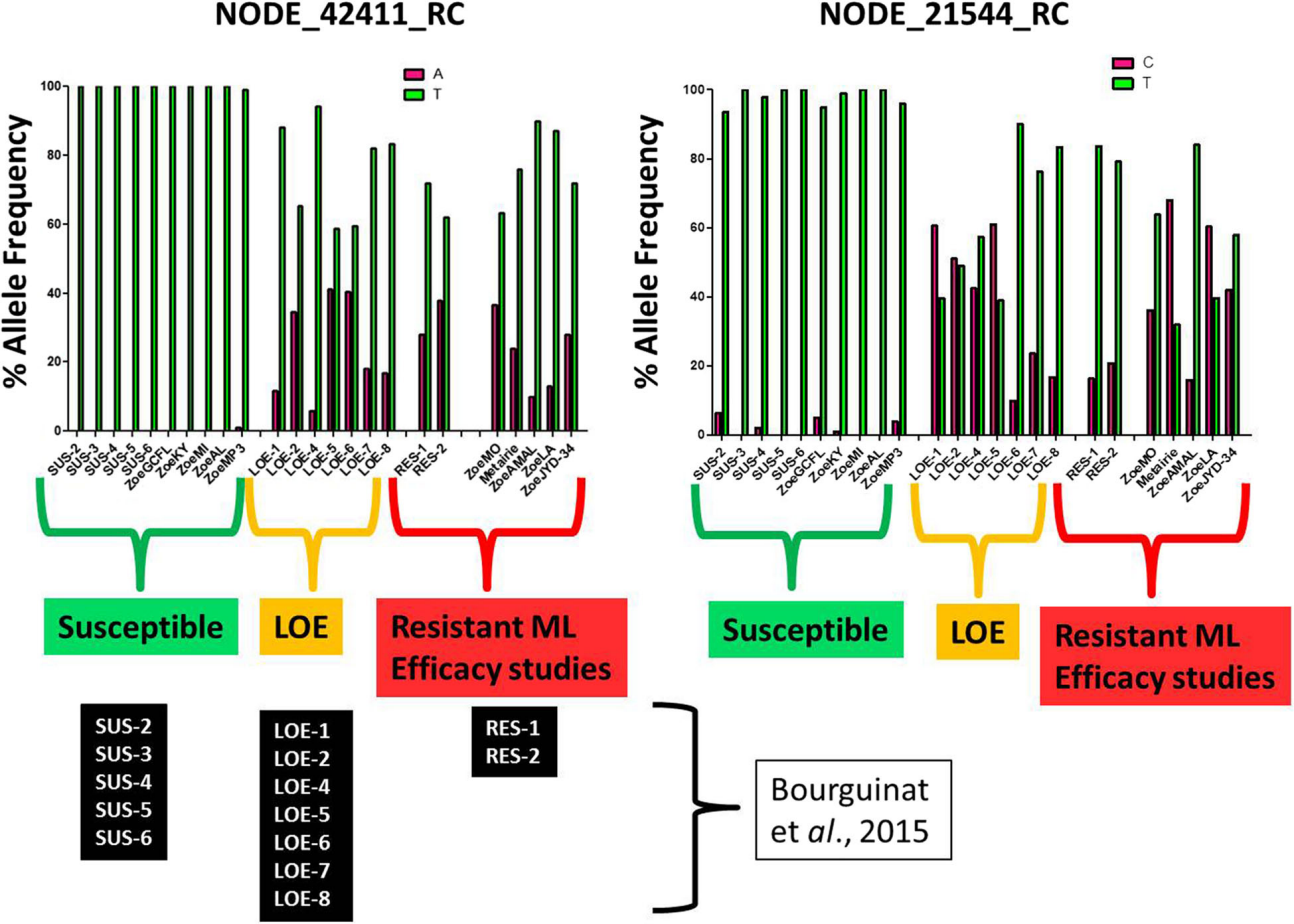
Example of two SNP positions (NODE_42411_RC and NODE_21544_RC) for which allele frequencies from ZoeSUS and ZoeRES are in accordance with previously reported data from ML-susceptible, LOE, and resistant isolates. RC stands for reverse complement

The mean depth sequencing coverage of the region, including the SNP, was ~2000X. The percentage frequencies of the alternative alleles of the 42 SNPs previously associated with LOE and resistance [13] are presented in Fig. 1. The differences of the percentage alternative allele frequencies between the new resistant isolates ZoeRES (ZoeMO, ZoeAMAL, ZoeLA, ZoeJYD-34, Metairie) and the new susceptible isolates ZoeSUS (ZoeKY, ZoeMI, ZoeGCFL, ZoeAL, ZoeMP3) showed that 40 of the 42 SNP positions had a higher percentage of the alternative alleles in ZoeRES compared with ZoeSUS (Fig. 1). The allele frequencies identified in the ZoeSUS and in ZoeRES populations are in accordance with the allele frequencies previously reported [13]. Two SNP positions (NODE_42411_RC and NODE_21554_RC) are presented as examples in Fig. 2 which shows the percentage allele frequencies of these two SNPs in all of the characterized *D. immitis* isolated collected so far. This current study allowed the number of SNPs that can best predict ML response in *D. immitis* to be narrowed.

**Fig. 3:**
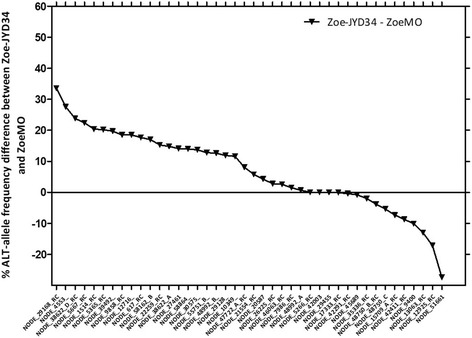
Difference in the percentage alternative allele frequencies of 42 SNPs between ZoeMO and ZoeJYD-34. RC stands for reverse complement

In addition, ZoeMO and ZoeJYD-34 isolates were compared. The difference between the two samples was that ZoeMO was the ZoeJYD-34 isolate re-passaged to a recipient dog 1.5 years later, with no intervening drug exposure. Thus, ZoeMO *D. immitis* population was a subset of the Zoe-JYD34 population (from a 50 L_3_ inoculum). So, ZoeMO and ZoeJYD-34 parasite populations are related but may not be genetically identical. ZoeMO and ZoeJYD-34 isolates had 82 and 19% percentage MOX efficacy, respectively [15] (Table 1). Interestingly, in Fig. 3, out the 42 SNPs described, 28 SNPs showed a higher frequency of the alternative allele (resistance associated) in ZoeJYD-34 compared with ZoeMO, six SNPs shared similar genetic profiles while nine SNPs had showed a higher frequency of the alternative allele in ZoeMO compared with ZoeJYD-34.

### Predictive model

**Fig. 4:**
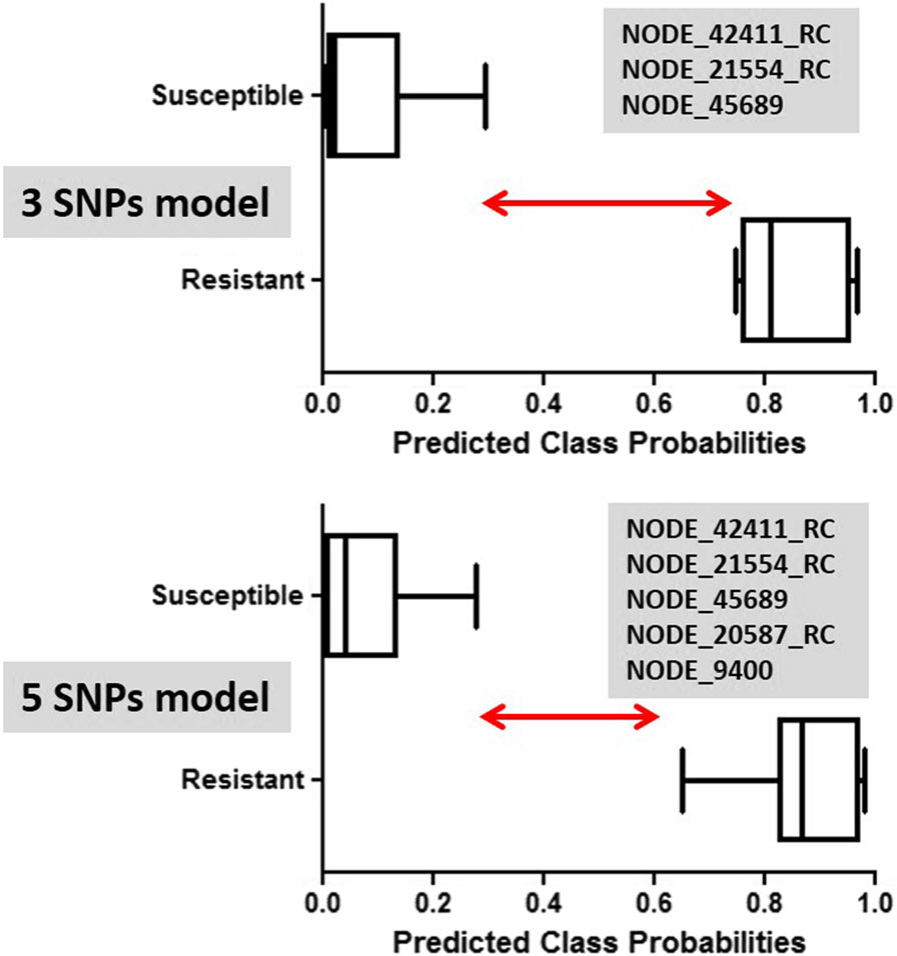
Mathematical models that predict for ML susceptibility and ML resistance in *D. immitis* isolates using combinations of three or five SNPs. The models were built with MetaboAnalyst 3.0 and Random Forest algorithm (*n* = 17). RC stands for reverse complement

**Fig. 5:**
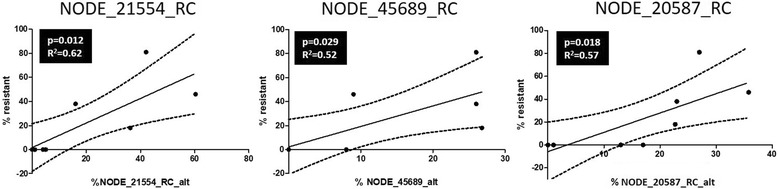
Linear regressions with some of the SNPs retained in the three-SNP and five-SNP models (Fig. 4). Each regression is based on the nine isolates for which efficacy data was available. RC and alt stand for reverse complement and alternative nucleotide, respectively

Using the Random Forest algorithm as implemented in the biomarker module from MetaboAnalyst 3.0, a series of predictive models were generated using any combination of two, three, five, and ten SNPs to differentiate the ML-susceptible isolates from the resistant isolates (see Additional file 3 at the end of this article). The results showed that using just a few SNPs, all models perform well with >90% sensitivity and 100% specificity. Given the relatively small the sample size (*n* = 17), it is expected that more robust performance estimate will be obtained when results of more isolates become available. For practical reasons for future field application, it was decided to test SNP combinations with the three- and five-SNP models, the three and five SNPs that gave, individually, the best performance (Fig. 4). Caution is necessary as they may be only the best individual SNP markers for this current dataset, and not necessarily for new isolates. Thus, there is a risk that performance evaluation was over-optimistic due to small sample size. With this in mind, only SNPs labeled NODE_42411_RC, NODE_21554_RC, and NODE_45689 were used in the three-SNP model. NODE_42411_RC, NODE_21554_RC, NODE_45689, NODE_20587_RC, and NODE_9400 were used in the five-SNP model. The results are presented in Fig. 4. Interestingly, the three-SNP model, using the SNPs NODE_42411_RC, NODE_21554_RC, and NODE_45689, better differentiated the ML-susceptible samples from the ML-resistant samples compared with the five-SNP model. In both cases, however, none of the samples were misclassified. When the allele frequencies of some of the SNPs used in the best three-SNP and five-SNP models were plotted against the percentage resistance (100 - % efficacy) for the nine isolates for which efficacy data are available, significant regressions were obtained (Fig. 5).

**Fig. 6:**
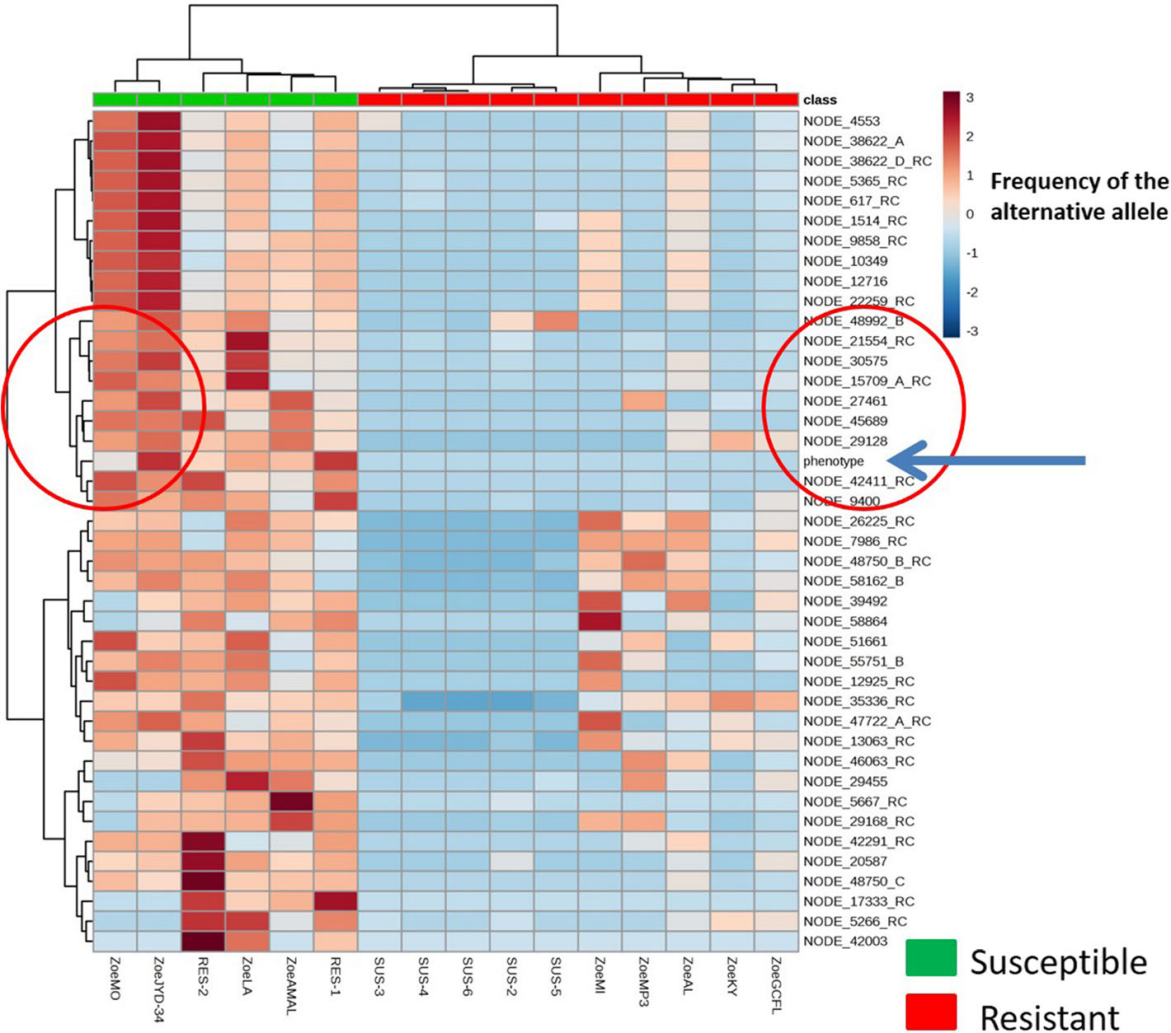
Heat map built with MetaboAnalyst 3.0 and Random Forest algorithm including genetic information and ML phenotype response from efficacy studies (*n* = 16). The red circles identified the SNPs that are the most correlated with ML phenotype response. RC stands for reverse complement

The heat map presented in Fig. 6 allowed the phenotype ML response of the isolates to be mapped against the percentage frequency of the alternative allele at all of the different SNP positions for all isolates with efficacy data. The analysis identified nine SNPs that were more closely correlated with the phenotype response such as NODE_42411_RC, NODE_9400, NODE_29128, NODE_45689, NODE_27461, NODE_15709_A_RC, NODE_30575, NODE_21554_RC, and NODE_48992_B. Some of the SNPs were identified earlier as having a better individual performance than others in the mathematical model (see Additional file 4 at the end of this article). The difference between the two analyses was that for the individual performance, the ML response was categorical (0 for susceptible and 1 for resistant), while in the heat map, phenotype was a continuous variable as the percentage reduction efficacy was used. Details of the estimation of efficacy are described in detail elsewhere [15]. Although the genetic data and the biological data were in accordance, the data should be treated with caution, as the efficacy studies and samples for genetic analysis (using untreated control dogs only) were related but not from the same dogs (in the efficacy studies, obviously the treated dogs were in the treatment group, whereas the MF for genetic analysis were from untreated dogs, from the same isolate). In general, the heat map showed significant differences in the frequencies of the alternative alleles between ML-susceptible and ML-resistant isolates.

## Discussion

At a time when ML resistance in *D. immitis* has become a concern [12, 13], identifying reliable genetic markers to predict ML response is important. The current study allowed us to determine the percentage of alternative alleles, previously identified as putative markers, in additional phenotypically well-characterized *D. immitis* isolates. ZoeRES isolates showed similar genetic profiles to LOE and confirmed resistant isolates previously reported [13], which was encouraging when seeking universal genetic markers to predict ML response in *D. immitis*. In addition, MiSeq technology appeared to be a suitable technology to work with MF pool samples.

Genetic analysis of ZoeMO and ZoeJYD-34 isolates was consistent with some reversion towards susceptibility in ZoeMO compared with parental ZoeJYD-34. Previous work *in Onchocerca volvulus* [29], a closely related filarial parasite, showed that female worms that carried an IVM selected genotype were less fertile than unselected worms. Thus, a possible difference in fitness between susceptible and resistant parasites could be considered. With no additional drug pressure on ZoeMO, the more susceptible female worms in the population may have produced more susceptible offspring (microfilariae) that could change the genetic profile and the resistance phenotype of ZoeMO compared with ZoeJYD-34. These fitness possibilities need investigation.

## Conclusion

Predictive models based on the Random Forest algorithm offer a promising approach to investigate which SNP combinations would best predict ML response. At the current stage, due to a limited sample size (*n* = 17), the combination of SNPs identified with the mathematical model may not be yet the final optimal set of markers. However, they will provide useful tools to consider when additional isolates can be added to the current models. This should help to identify a small number of the SNP for field monitoring for resistance. A new study supported by the American Heartworm Society to further evaluate the SNP markers in isolates coming from veterinary clinics in U.S., should provide additional information and increase confidence in using these SNPs for resistance identification and monitoring.

## Additional files

Additional file 1: SNP localization in *Dirofilaria immitis* genome. List of the SNPs that were investigated, including the position of the SNPs in the scaffold of the *D. immitis* genome nDi.2.2. (http://www.nematodes.org/genomes/dirofilaria_immitis/). (DOCX 16 kb)

Additional file 2: List of primers sets for MiSeq. Forward primers were composed of CS1 primer + specific sequences while reverse primers were composed of CS2 primer + specific sequences. (XLSX 15 kb)

Additional file 3: Mathematical models that used the biomarker tools from MetaboAnalyst 3.0 with the Random Forest algorithm to predict macrocyclic lactone susceptibility or resistance in *Dirofilaria immitis* isolates*.* Four models are presented based on any combinations of two, three, five, or ten SNPs. The samples (*n* = 17) contained ten susceptible samples (ZoeAL, ZoeGCFL, ZoeKY, ZoeMI, ZoeMP3 from the current study, and SUS-2, SUS-3, SUS-4, SUS-5, SUS-6 from Bourguinat et al. [13]), and seven resistant samples (Metairie, ZoeAMAL, ZoeJYD-34, ZoeLA, ZoeMO from the current study, and RES-1, RES-2 from Bourguinat et al. [13]). The results are presented in box plot format. Zero was the optimal value for macrocyclic lactone susceptibility prediction. One was the optimal value for macrocyclic lactone resistance prediction. A cut-off at 0.5 was set, which meant that any sample with a predicted class probability less than 0.5 was considered as macrocyclic lactone susceptible while any sample with a predicted class probability higher than 0.5 was considered as macrocyclic lactone resistant. These models allowed identification of the sensitivity [True Positive/(True Positive + False Negative)] and the specificity [True Negative/(False Positive + True Negative)] of the difference. In the two- or three-SNP models, ZoeAL (susceptible) was closest to the cut-off of 0.5 (0.47 and 0.49, respectively). However, in the five- and ten-SNP models, ZoeAL appeared as a false positive (false resistant) (0.52 and 0.54, respectively). The current result should be taken with caution due to sample size, but this analysis shows the potential of using mathematical modeling to identify the best SNP combinations using larger sample size. (DOCX 108 kb)

Additional file 4: Individual SNP marker performance identified with MetaboAnalyst using the Random Forest algorithm. The performance was calculated based on 17 well-characterized samples in term of macrocyclic lactone responses: ten susceptible samples (ZoeAL, ZoeGCFL, ZoeKY, ZoeMI, ZoeMP3 from the current study and SUS-2, SUS-3, SUS-4, SUS-5, SUS-6 from Bourguinat et al. [13] and seven resistant samples (Metairie, ZoeAMAL, ZoeJYD-34, ZoeLA, ZoeMO from the current study and RES-1, RES-2 from Bourguinat et al. [13]. Caution is indicated as SNP markers may be only sorted in terms of performance due to this particular dataset, but the order may be different with new samples. Thus, there is a risk that the current performance evaluation is over-optimistic. RC stands for reverse complement. (DOCX 15 kb)

## Abbreviations

IVM: Ivermectin
MF: Microfilaria
ML: Macrocyclic lactone
MOX: Moxidectin

## References

[1] Bowman DD, Atkins CE (2009). Heartworm biology, treatment, and control. Vet Clin North Am Small Anim Pract.

[2] Carlson BLN, Nielsen SW (1985). Vena caval syndrome in a coyote. Vet Med.

[3] Kreeger TJ, Seal US, Callahan M, Beckel M (1990). Treatment and prevention with ivermectin of dirofilariasis and ancylostomiasis in captive gray wolves (*Canis lupus*). J Zoo Wildlife Med..

[4] Miller DL, Schrecengost J, Kilgo J, Ray HS, Miller KV (2007). Ruptured aortic aneurysm in a coyote (Canis Latrans) from South Carolina. J Zoo Wildlife Med.

[5] Phillips MK, Scheck J (1991). Parasitism in captive and reintroduced red wolves. J Wildlife Dis.

[6] Pratt SE, Mall JJ, Rhoades JD, Hertzog RE, Corwin RM (1981). Dirofilariasis in a captive wolf pack. Vet Med Small Anim Clin.

[7] Aranda C, Panyella O, Eritja R, Castella J (1998). Canine filariasis. Importance and transmission in the Baix Llobregat area, Barcelona (Spain). Vet Parasitol.

[8] Soulsby EJL, Mönnig HO (1968). Helminths, arthropods, & protozoa of domesticated animals: Baillière, Tindall & Cassell.

[9] Bowman DD, Mannella C (2011). Macrocyclic lactones and *Dirofilaria immitis* microfilariae. Top Comp Anim Med.

[10] Hampshire VA (2005). Evaluation of efficacy of heartworm preventive products at the FDA. Vet Parasitol.

[11] Atkins CE, Murray MJ, Olavessen LJ, Burton KW, Marshall JW, Brooks CC (2014). Heartworm ‘lack of effectiveness’ claims in the Mississippi delta: computerized analysis of owner compliance--2004-2011. Vet Parasitol.

[12] Pulaski CN, Malone JB, Bourguinat C, Prichard R, Geary T, Ward D, Klei TR, Guidry T, Smith G, Delcambre B (2014). Establishment of macrocyclic lactone resistant Dirofilaria immitis isolates in experimentally infected laboratory dogs. Parasit Vectors.

[13] Bourguinat C, Lee AC, Lizundia R, Blagburn BL, Liotta JL, Kraus MS, Keller K, Epe C, Letourneau L, Kleinman CL (2015). Macrocyclic lactone resistance in Dirofilaria immitis: failure of heartworm preventives and investigation of genetic markers for resistance. Vet Parasitol.

[14] Prichard R (2001). Genetic variability following selection of Haemonchus contortus with anthelmintics. Trends Parasitol.

[15] McTier T, Six R, Pullins A, Chapin S, McCall J, Rugg D, Maeder SJ, Woods DJ. Efficacy of oral moxidectin against susceptible and resistant isolates of Dirofilaria immitis in dogs. Parasit Vectors. 2017;10(Suppl 2): doi:10.1186/s13071-017-2429-5. 10.1186/s13071-017-2429-5 PMC5688394 29143634

[16] Maclean MJ, Molly D, Savadelis MD, Coates R, Dzimiansky M, Jones C, Benbow C, Kaplan RM, Moorhead AR, Wolstenholme AJ. Does evaluation of in vitro microfilarial motility reflect the resistance status of Dirofilaria immitis isolates to macrocyclic lactones? Parasit Vectors. 2017;10(Suppl 2): doi:10.1186/s13071-017-2436-6. 10.1186/s13071-017-2436-6 PMC5688452 29143656

[17] Ison SA, Delannoy S, Bugarel M, Nagaraja TG, Renter DG, den Bakker HC, Nightingale KK, Fach P, Loneragan GH (2016). Targeted amplicon sequencing for single-nucleotide-polymorphism genotyping of attaching and effacing Escherichia Coli O26:H11 cattle strains via a high-throughput library preparation technique. Appl Environ Microbiol.

[18] Bronner IF, Quail MA, Turner DJ, Swerdlow H (2014). Improved protocols for illumina sequencing. Curr Protoc Hum Genet.

[19] Bolger AM, Lohse M, Usadel B (2014). Trimmomatic: a flexible trimmer for Illumina sequence data. Bioinformatics.

[20] Li H, Durbin R (2010). Fast and accurate long-read alignment with burrows-wheeler transform. Bioinformatics.

[21] Ho TK (1998). The random subspace method for constructing decision forests. Ieee T Pattern Analysis.

[22] Xia J, Psychogios N, Young N, Wishart DS (2009). MetaboAnalyst: a web server for metabolomic data analysis and interpretation. Nucleic Acids Res.

[23] Xia J, Wishart DS (2011). Web-based inference of biological patterns, functions and pathways from metabolomic data using MetaboAnalyst. Nat Protoc.

[24] Xia J, Wishart DS (2011). Metabolomic data processing, analysis, and interpretation using MetaboAnalyst. Curr Protoc Bioinformatics.

[25] Xia J, Mandal R, Sinelnikov IV, Broadhurst D, Wishart DS (2012). MetaboAnalyst 2.0--a comprehensive server for metabolomic data analysis. Nucleic Acids Res.

[26] Xia J, Sinelnikov IV, Han B, Wishart DS (2015). MetaboAnalyst 3.0--making metabolomics more meaningful. Nucleic Acids Res.

[27] Bureau A, Dupuis J, Falls K, Lunetta KL, Hayward B, Keith TP, Van Eerdewegh P (2005). Identifying SNPs predictive of phenotype using random forests. Genet Epidemiol.

[28] Chen X, Ishwaran H (2012). Random forests for genomic data analysis. Genomics.

[29] Bourguinat C, Pion SD, Kamgno J, Gardon J, Gardon-Wendel N, Duke BO, Prichard RK, Boussinesq M (2006). Genetic polymorphism of the beta-tubulin gene of *Onchocerca volvulus* in ivermectin naive patients from Cameroon, and its relationship with fertility of the worms. Parasitology.

