# Supplementary material for: Genetic profiles of ten Dirofilaria immitis isolates susceptible or resistant to macrocyclic lactone heartworm preventives
Source: Parasit Vectors. 2017 Nov 9;10(Suppl 2):504. doi: 10.1186/s13071-017-2428-6 (PMC5688420; doi:10.1186/s13071-017-2428-6)
Supplement: Supplementary file 1 — SNP localization in Dirofilaria immitis genome. List of the SNPs that were investigated, including the position of the SNPs in the scaffold of the D. immitis genome nDi.2.2. (http://www.nematodes.org/genomes/dirofilaria_immitis/). (DOCX 16 kb) [file 13071_2017_2428_MOESM1_ESM.docx]

**Additional file 1:** SNP localization in *Dirofilaria immitis* genome

| **SNP** | **ID** | **Scaffold** | **Position** | **SNP** | **ID** | **Scaffold** | **Position** |
| --- | --- | --- | --- | --- | --- | --- | --- |
| **1** | NODE_42411 | nDi.2.2.scaf00001 | 466197 | **22** | NODE_20587 | nDi.2.2.scaf00185 | 62174 |
| **2** | NODE_42291 | nDi.2.2.scaf00001 | 748216 | **23** | NODE_5266 | nDi.2.2.scaf00215 | 28260 |
| **3** | NODE_48992_A | nDi.2.2.scaf00004 | 79159 | **24** | NODE_48750_C | nDi.2.2.scaf00238 | 10209 |
| **4** | NODE_48992_B | nDi.2.2.scaf00004 | 79766 | **25** | NODE_48750_B | nDi.2.2.scaf00238 | 29165 |
| **5** | NODE_9400 | nDi.2.2.scaf00005 | 662854 | **26** | NODE_42003 | nDi.2.2.scaf00284 | 33605 |
| **6** | NODE_10349 | nDi.2.2.scaf00007 | 300005 | **27** | NODE_46063 | nDi.2.2.scaf00284 | 42920 |
| **7** | NODE_9858 | nDi.2.2.scaf00007 | 375510 | **28** | NODE_26225 | nDi.2.2.scaf00293 | 46955 |
| **8** | NODE_1514 | nDi.2.2.scaf00019 | 442063 | **29** | NODE_29455 | nDi.2.2.scaf00377 | 15477 |
| **9** | NODE_12716 | nDi.2.2.scaf00021 | 25243 | **30** | NODE_58162_B | nDi.2.2.scaf00492 | 12704 |
| **10** | NODE_22259 | nDi.2.2.scaf00021 | 212599 | **31** | NODE_5667 | nDi.2.2.scaf00495 | 19924 |
| **11** | NODE_5365 | nDi.2.2.scaf00021 | 379745 | **32** | NODE_55751_B | nDi.2.2.scaf00582 | 14587 |
| **12** | NODE_617 | nDi.2.2.scaf00021 | 387398 | **33** | NODE_35336 | nDi.2.2.scaf00589 | 15334 |
| **13** | NODE_47722_A | nDi.2.2.scaf00023 | 336087 | **34** | NODE_29128 | nDi.2.2.scaf00597 | 12915 |
| **14** | NODE_30575 | nDi.2.2.scaf00046 | 22857 | **35** | NODE_39492 | nDi.2.2.scaf00664 | 25005 |
| **15** | NODE_15709_A | nDi.2.2.scaf00046 | 76278 | **36** | NODE_7986 | nDi.2.2.scaf00669 | 3266 |
| **16** | NODE_21554 | nDi.2.2.scaf00046 | 222254 | **37** | NODE_12925 | nDi.2.2.scaf00706 | 13761 |
| **17** | NODE_38622_A | nDi.2.2.scaf00056 | 97452 | **38** | NODE_17333 | nDi.2.2.scaf01340 | 1522 |
| **18** | NODE_38622_D | nDi.2.2.scaf00056 | 130632 | **39** | NODE_13063 | nDi.2.2.scaf01422 | 4176 |
| **19** | NODE_4553 | nDi.2.2.scaf00107 | 106233 | **40** | NODE_29168 | nDi.2.2.scaf01527 | 5968 |
| **20** | NODE_27461 | nDi.2.2.scaf00140 | 30919 | **41** | NODE_58864 | nDi.2.2.scaf06378 | 423 |
| **21** | NODE_45689 | nDi.2.2.scaf00185 | 10639 | **42** | NODE_51661 | nDi.2.2.scaf06614 | 182 |

List of the SNPs that were investigated including the position of the SNPs in the scaffold of the *D. immitis* genome nD.2.2. (http://www.nematodes.org/genomes/dirofilaria_immitis/)
