# Supplementary material for: Genetic profiles of ten Dirofilaria immitis isolates susceptible or resistant to macrocyclic lactone heartworm preventives
Source: Parasit Vectors. 2017 Nov 9;10(Suppl 2):504. doi: 10.1186/s13071-017-2428-6 (PMC5688420; doi:10.1186/s13071-017-2428-6)

**Additional file 3:** Mathematical models that used the biomarker tools from Metaboanalyst 3.0 with Random Forest algorithm to predict macrocyclic lactone susceptibility or resistance in *Dirofilaria immitis.* Four models are presented based on any combinations of 2, 3, 5, or 10 SNPs. The samples (n=17) contained 10 susceptible samples (ZoeAL, ZoeGCF, ZoeKY, ZoeMI, ZoeMP3 from the current study and SUS-2, SUS-3, SUS-4, SUS-5, SUS-6 from Bourguinat et al. [13]) and 7 resistant samples (Metairie, ZoeAMAL, ZoeJYD-34, ZoeLA, ZoeMO from the current study and RES-1, RES-2 from Bourguinat et al. [13]). The results are presented in box plot format. Zero was the optimal value for macrocyclic lactone susceptibility prediction. One was the optimal value for macrocyclic lactone resistance prediction. A cut-off at 0.5 was set, which meant that any samples with a predicted class probability less than 0.5 was considered as macrocyclic lactone susceptible while any samples with a predicted class probability higher than 0.5 was considered as macrocyclic lactone resistant. These models allowed identification of sensitivity (True Positive/ (True Positive + False Negative)) and the specificity (True Negative/(False Positive + True Negative)) of the difference. In the 2 or 3 SNPs models, ZoeAL (susceptible) was closest to the cut-off of 0.5 (0.47 and 0.49, respectively). However, in the 5 and 10 SNPs models, ZoeAL appeared as a False Positive (False resistant) (0.52 and 0.54 respectively). The current result should be taken with caution due to sample size, but this analysis shows the potential of using mathematical modeling to identify the best SNP combinations using larger sample size.


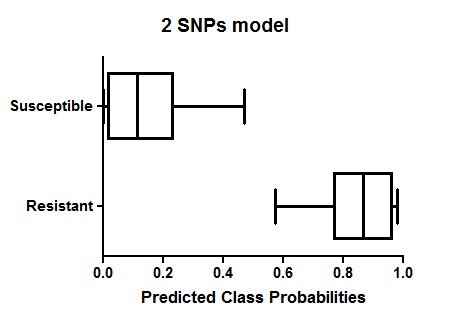

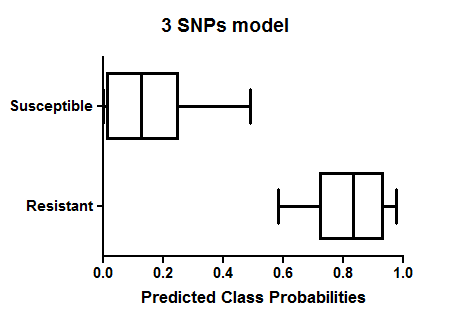

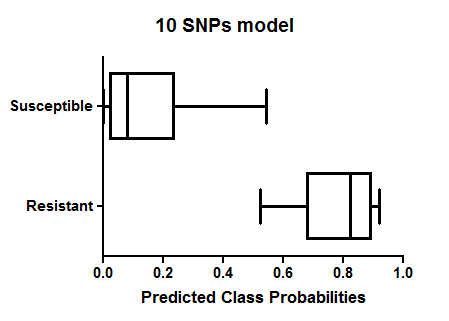

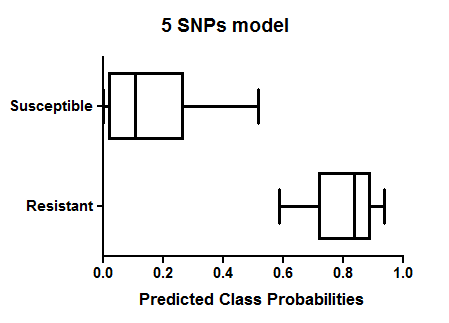

Supplement: Supplementary file 3 — Mathematical models that used the biomarker tools from MetaboAnalyst 3.0 with the Random Forest algorithm to predict macrocyclic lactone susceptibility or resistance in Dirofilaria immitis isolates. Four models are presented based on any combinations of two, three, five, or ten SNPs. The samples (n = 17) contained ten susceptible samples (ZoeAL, ZoeGCFL, ZoeKY, ZoeMI, ZoeMP3 from the current study, and SUS-2, SUS-3, SUS-4, SUS-5, SUS-6 from Bourguinat et al. [13]), and seven resistant samples (Metairie, ZoeAMAL, ZoeJYD-34, ZoeLA, ZoeMO from the current study, and RES-1, RES-2 from Bourguinat et al. [13]). The results are presented in box plot format. Zero was the optimal value for macrocyclic lactone susceptibility prediction. One was the optimal value for macrocyclic lactone resistance prediction. A cut-off at 0.5 was set, which meant that any sample with a predicted class probability less than 0.5 was considered as macrocyclic lactone susceptible while any sample with a predicted class probability higher than 0.5 was considered as macrocyclic lactone resistant. These models allowed identification of the sensitivity [True Positive/(True Positive + False Negative)] and the specificity [True Negative/(False Positive + True Negative)] of the difference. In the two- or three-SNP models, ZoeAL (susceptible) was closest to the cut-off of 0.5 (0.47 and 0.49, respectively). However, in the five- and ten-SNP models, ZoeAL appeared as a false positive (false resistant) (0.52 and 0.54, respectively). The current result should be taken with caution due to sample size, but this analysis shows the potential of using mathematical modeling to identify the best SNP combinations using larger sample size. (DOCX 108 kb) [file 13071_2017_2428_MOESM3_ESM.docx]
