# Supplementary material for: Genetic profiles of ten Dirofilaria immitis isolates susceptible or resistant to macrocyclic lactone heartworm preventives
Source: Parasit Vectors. 2017 Nov 9;10(Suppl 2):504. doi: 10.1186/s13071-017-2428-6 (PMC5688420; doi:10.1186/s13071-017-2428-6)
Supplement: Supplementary file 4 — Individual SNP marker performance identified with MetaboAnalyst using the Random Forest algorithm. The performance was calculated based on 17 well-characterized samples in term of macrocyclic lactone responses: ten susceptible samples (ZoeAL, ZoeGCFL, ZoeKY, ZoeMI, ZoeMP3 from the current study and SUS-2, SUS-3, SUS-4, SUS-5, SUS-6 from Bourguinat et al. [13] and seven resistant samples (Metairie, ZoeAMAL, ZoeJYD-34, ZoeLA, ZoeMO from the current study and RES-1, RES-2 from Bourguinat et al. [13]. Caution is indicated as SNP markers may be only sorted in terms of performance due to this particular dataset, but the order may be different with new samples. Thus, there is a risk that the current performance evaluation is over-optimistic. RC stands for reverse complement. (DOCX 15 kb) [file 13071_2017_2428_MOESM4_ESM.docx]

**Additional file 4:** Individual SNP marker performance identified with Metaboanalyst using the Random Forest algorithm. The performance was calculated based on 17 well characterized samples in term of macrocyclic lactone responses, 10 susceptible samples (ZoeAL, ZoeGCF, ZoeKY, ZoeMI, ZoeMP3from the current study and SUS-2, SUS-3, SUS-4, SUS-5, SUS-6 from Bourguinat et al. (2015) and 7 resistant samples (Metairie, ZoeAMAL, ZoeJYD-34, ZoeLA, ZoeMO from the current study and RES-1, RES-2 from Bourguinat et al. (2015). Caution is indicated, as SNP markers may be only sorted in term of performance due to this particular dataset, but the order may be different with new samples. Thus, there is a risk that the current performance evaluation is over-optimistic. RC stands for reverse complement

| **Name** | **AUC** | **T-tests** | **Fold Change** |
| --- | --- | --- | --- |
| NODE_42411_RC | 1 | 1.84E-06 | -7.4131 |
| NODE_21554_RC | 1 | 8.74E-05 | -5.7923 |
| NODE_45689 | 1 | 2.61E-06 | -7.0045 |
| NODE_20587 | 1 | 5.54E-05 | -5.7803 |
| NODE_9400 | 0.98571 | 4.31E-06 | -5.7681 |
| NODE_30575 | 0.98571 | 9.14E-05 | -6.4588 |
| NODE_10349 | 0.97143 | 3.46E-04 | -5.9938 |
| NODE_617_RC | 0.97143 | 3.97E-04 | -6.3583 |
| NODE_38622_A | 0.97143 | 2.09E-04 | -5.9788 |
| NODE_15709_A_RC | 0.96429 | 2.83E-04 | -5.8998 |
| NODE_38622_D_RC | 0.96429 | 0.001072 | -6.4845 |
| NODE_9858_RC | 0.95714 | 5.04E-04 | -5.9623 |
| NODE_12716 | 0.95714 | 3.69E-04 | -5.7751 |
| NODE_22259_RC | 0.95714 | 2.48E-04 | -6.0542 |
| NODE_5365_RC | 0.95714 | 4.71E-04 | -6.3144 |
| NODE_27461 | 0.95714 | 2.07E-04 | -6.1466 |
| NODE_42291_RC | 0.95 | 0.001719 | -5.4518 |
| NODE_4553_ | 0.94286 | 7.48E-04 | -5.861 |
| NODE_12925_RC | 0.92857 | 1.86E-04 | -7.4256 |
| NODE_17333_RC | 0.92857 | 0.010748 | -5.1454 |
| NODE_48992_B_ | 0.91429 | 9.47E-04 | -4.9 |
| NODE_48750_C | 0.91429 | 0.001846 | -5.3791 |
| NODE_51661 | 0.9 | 0.003074 | -4.9379 |
| NODE_47722_A_RC | 0.88571 | 0.005457 | -4.7668 |
| NODE_46063_RC | 0.88571 | 0.002051 | -6.0821 |
| NODE_29128 | 0.87857 | 0.003312 | -5.0469 |
| NODE_58162_B | 0.87143 | 0.005937 | -3.8346 |
| NODE_55751_B | 0.87143 | 0.006156 | -5.4865 |
| NODE_5667_RC | 0.86429 | 0.005302 | -4.7312 |
| NODE_13063_RC | 0.83571 | 0.014453 | -3.7041 |
| NODE_1514_RC | 0.82143 | 0.0153 | -3.9177 |
| NODE_48750_B_RC | 0.81429 | 0.02538 | -3.4535 |
| NODE_35336_RC | 0.80714 | 0.014422 | -2.9993 |
| NODE_7986_RC | 0.8 | 0.060487 | -4.7216 |
| NODE_26225_RC | 0.77143 | 0.04435 | -3.9576 |
| NODE_29455 | 0.77143 | 0.021439 | -3.2653 |
| NODE_29168_RC | 0.76429 | 0.029949 | -3.6433 |
| NODE_39492 | 0.72857 | 0.24781 | -3.5549 |
| NODE_42003 | 0.71429 | 0.045787 | -3.4043 |
| NODE_58864 | 0.7 | 0.3079 | -3.3168 |
| NODE_5266_RC | 0.64286 | 0.066037 | -1.4254 |
